# An American Text-Book of Diseases of the Eye, Ear, Nose and Throat

**Published:** 1899-06

**Authors:** 


AN AMERICAN TEXT-BOOK OF DISEASES OF THE EYE, EAR, NOSE AND THROAT. Edited by G. E. DE SCHWEINITZ, A. M., M. D., Professor of Ophthalmology in the Jefferson Medical College, Philadelphia; Consulting Ophthalmologist to the Philadelphia Polyclinic; Ophthalmic Surgeon to the Philadelphia Hospital and to the Orthopedic Hospital and Infirmary for Nervous Diseases; and B. ALEX. RANDALL, M. A., M. D., Ph. D., Clinical Professor of Diseases of the Ear in the University of Pennsylvania; Professor of Diseases of the Ear in the Philadelphia Polyclinic; Ophthalmic and Aural Surgeon to the Methodist and Children's Hospitals, Philadelphia. Illustrated with 766 engravings, fifty-nine of them in colors; 1251 pages. Philadelphia: W. B. Saunders. 1899.

This volume is another valuable accession to the series of American text-books which W. B. Saunders has been producing during the past few years with so much favor. The editors have comprised within the single volume, diseases of the eye, ear, nose and throat, the propriety of which is suggested by the fact that these organs are more or less anatomically related to each other, and have many points of physiological analogy. As Dr. D. H. Knapp, of New York, has said, "the physician who has trained himself in the diagnosis of the affections of one of these organs is prepared to master the methods of examination of the others. The four constitute a well rounded field of labor, which is not at all narrow, yet much more restricted than general surgery."

The "collaboration" method has been adopted, each section being contributed by an author especially fitted by study and practice to treat the subject assigned to him. Sixty authors have thus been drawn upon, and the result has been a most satisfactory and complete handbook. By reason of the exacting editorial supervision and direction, there is very little over-lapping or repetition of matter, and a reasonable proportion between the sections, commensurate with the importance of the subjects, is maintained throughout.

In the part devoted to the eye the subjects have been assigned as follows: embryology, anatomy and histology, Dr. G. A. Piersol; physiology of vision, Dr. A. P. Brubaker; optics, Drs. W. S. Dennett and C. W. Cutter; examination, Dr. G. E. de Schweinitz; the ophthalmoscope, Dr. B. A. Randall; determination and correction of refraction, Dr. E. Jackson; spectacles, Dr. R. J. Phillips; diseases of the eye lids, Dr. B. L. Millikin; diseases of the lachrymal apparatus, Dr. S. Theobold; diseases of the conjunctiva, Dr. J. E. Weeks; diseases of the cornea and sclera, Dr. S. M. Burnett; diseases of the iris, ciliary body and choroid and sympathetic inflammation and irritation, Dr. R. L. Randolph; injuries of the eye and its appendages, Dr. A. A. Hubbell; glaucoma, Dr. J. A. Lippincott; diseases of the lens, Dr. W. E. Hopkins; diseases of the vitreous humor, Dr. F. Carrow; diseases of the retina, Dr. L. Howe; diseases of the optic nerve, Dr. H. Gifford; amblyopia, Dr. C. A. Wood; partial amblyopias, Dr. H. V. Würdemann; intraocular growths, Dr. W. A. Holden; movements of the eyeballs and their anomalies, Dr. A. Duane; injuries and diseases of the orbit, Dr. F. Buller; operations: preparation, Dr. G. E. de Schweinitz; on eyelids, Dr. F. C. Hotz; on conjunctiva, cornea, sclera, and for evisceration and enucleation, Dr. C. W. Kollock; on iris and lens, Dr. H. Knapp; on eye muscles, Dr. S. C. Ayres; on lachrymal apparatus, Dr. S. Theobold; on the orbit, Dr. F. Buller. An appendix to this part includes, detection of color-blindness, Dr. J. E. Jennings, and Dr. A. G. Thomson; Röntgen rays in ophthalmic surgery, Dr. W. M. Sweet; practice of ophthalmic operations on animals' eyes, Dr. C. A. Veasey; microörganisms in ocular diseases, Dr. G. E. de Schweinitz.

In the second part, on the ear, its anatomy, embryology and histology are contributed by Dr. B. A. Randall, physiology, Dr. F. Allport; etiology and pathology, Dr. C. R. Holmes; examination, symptomatology and diagnosis, Dr. J. E. Sheppard; general therapeutics, Dr. C. E. Blake; diseases of the external ear, Dr. S. Theobold; injuries and diseases of the drumhead, Dr. H. V. Würdemann acute affections of the tympanic cavity and Eustachian tube, Dr. H. G. Miller; chronic catarrh of the middle ear, Dr. E. B. Dench; chronic suppuration of the middle ear, Dr. A. H. Buck; complications of tympanic inflammation, Dr. H. Knapp; diseases of the sound-perceiving apparatus, Dr. H. A. Alderton; operations, Dr. J. O. Green.

Part third, includes diseases of the nose and throat, and the contributions are as follows: anatomy, histology and embryology of the upper air passages, Drs. H. Allen and A. A. Bliss; physiology, Dr. W. J. Freeman; etiology and pathology, Dr. J. H. Bryan; examination and diagnosis, Dr. J. W. Farlow; therapeusis and prognosis, Dr. G. A. Leland; acute affections of the nose, Dr. W. E. Casselberry; chronic affections of the nose; Dr. M. J. Asch; diseases of the tonsils, palate and pharynx, Dr. J. E. Newcomb; atrophic rhinitis, Dr. W. P. Porcher; diseases of the sinuses, Dr. R. C. Myles; acute affections of the larynx and trachea, Dr. W. E. Hopkins; chronic inflammations of the larynx, Dr. C. E. de M. Sajous; diphtheria, Dr. J. H. McCollom; tuberculosis, Dr. E. L. Shurly; syphilis, Dr. W. C. Glasgow ; neoplasms, Dr. J. Wright; injuries and deformities, Dr. J. O. Roe; neuroses, Dr. J. Wright; the voice, Dr. G. H. Makuen; operations, Dr. J. O. Roe.

A survey of the above list of authors, most of whom are well-known to the profession, is a sufficient guarantee of the reliability of the work and of the thoroughness with which it has been done. The illustrations and press-work are of superior excellence. Taken altogether, there is nothing in the English language that can excel this work as a textbook on the subjects of which it treats, both in its completeness and trustworthiness. A. A. H.

